# Supplementary material for: Phylogenetic Revision and Patterns of Host Specificity in the Fungal Subphylum Entomophthoromycotina
Source: Microorganisms. 2022 Jan 24;10(2):256. doi: 10.3390/microorganisms10020256 (PMC8879804; doi:10.3390/microorganisms10020256)
Supplement: Supplementary file 1 [file microorganisms-10-00256-s001.zip › Supplementary Figures S1-S5.pdf]

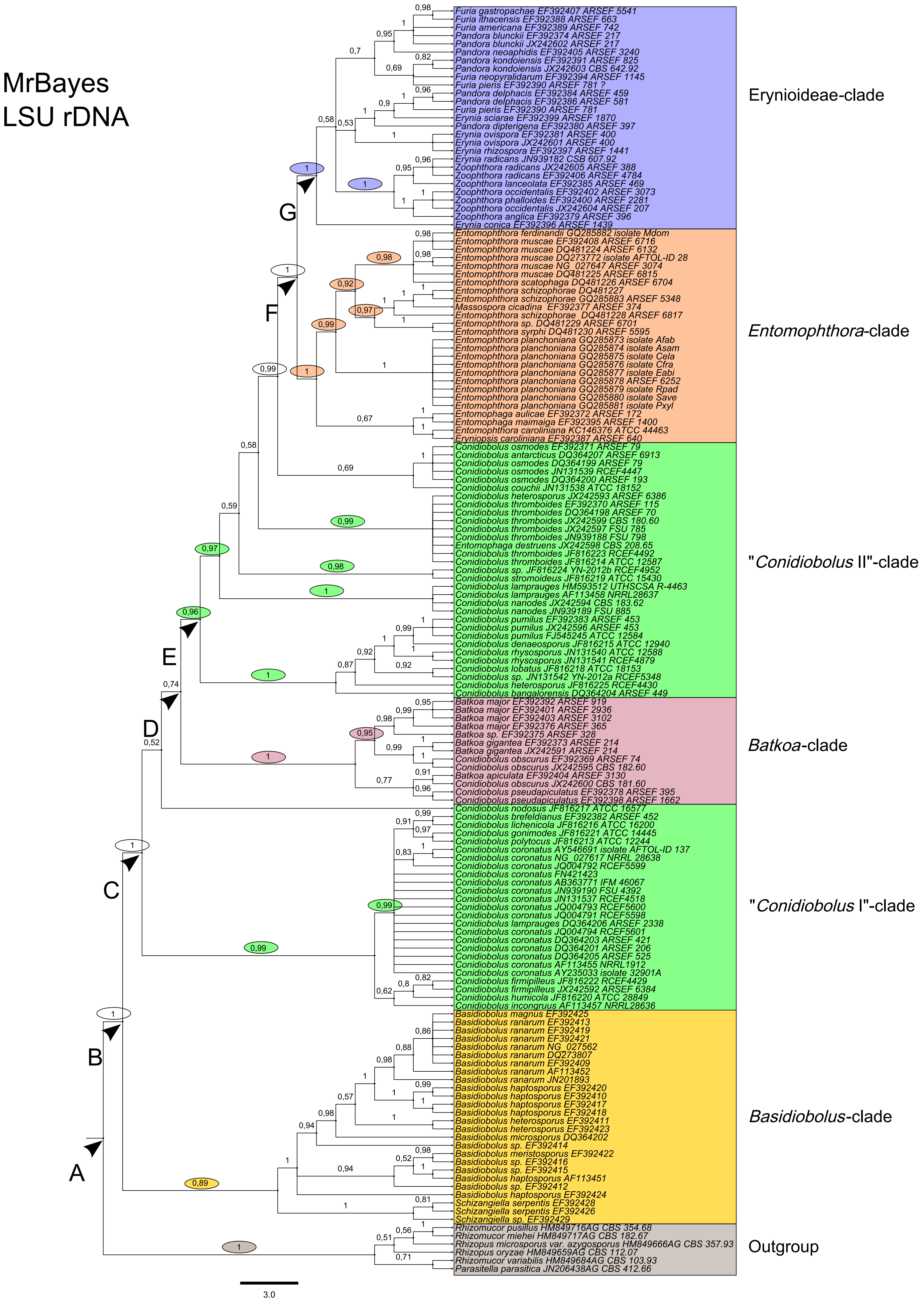

Figure S1: Entomophthoromycotina phylogeny based on a bayesian phylogram reconstruction inferred from large subunit (28S, LSU) ribosomal RNA nucleotide sequences of 147 strains. High, well, and moderate statistical bootstrap support (BS, inferred with MrBayes on a scale from 0 to 1) is indicated above branches in the color code of the corresponding clade where applicable as in Fig. 3. (A) Split of the outgroup from the Entomophthoromycota lineage, (B) Split of the *Basidiobolus*-clade, (C) Split of the "*Conidiobolus* I"-clade, (D) Split of the *Batkoa*-clade, (E) Split of the "*Conidiobolus* II"-clade, (F) Split of the *Entomophthora*-clade, (G) Split of the Erynioideae-clade. Scale bar indicates substitutions per site.

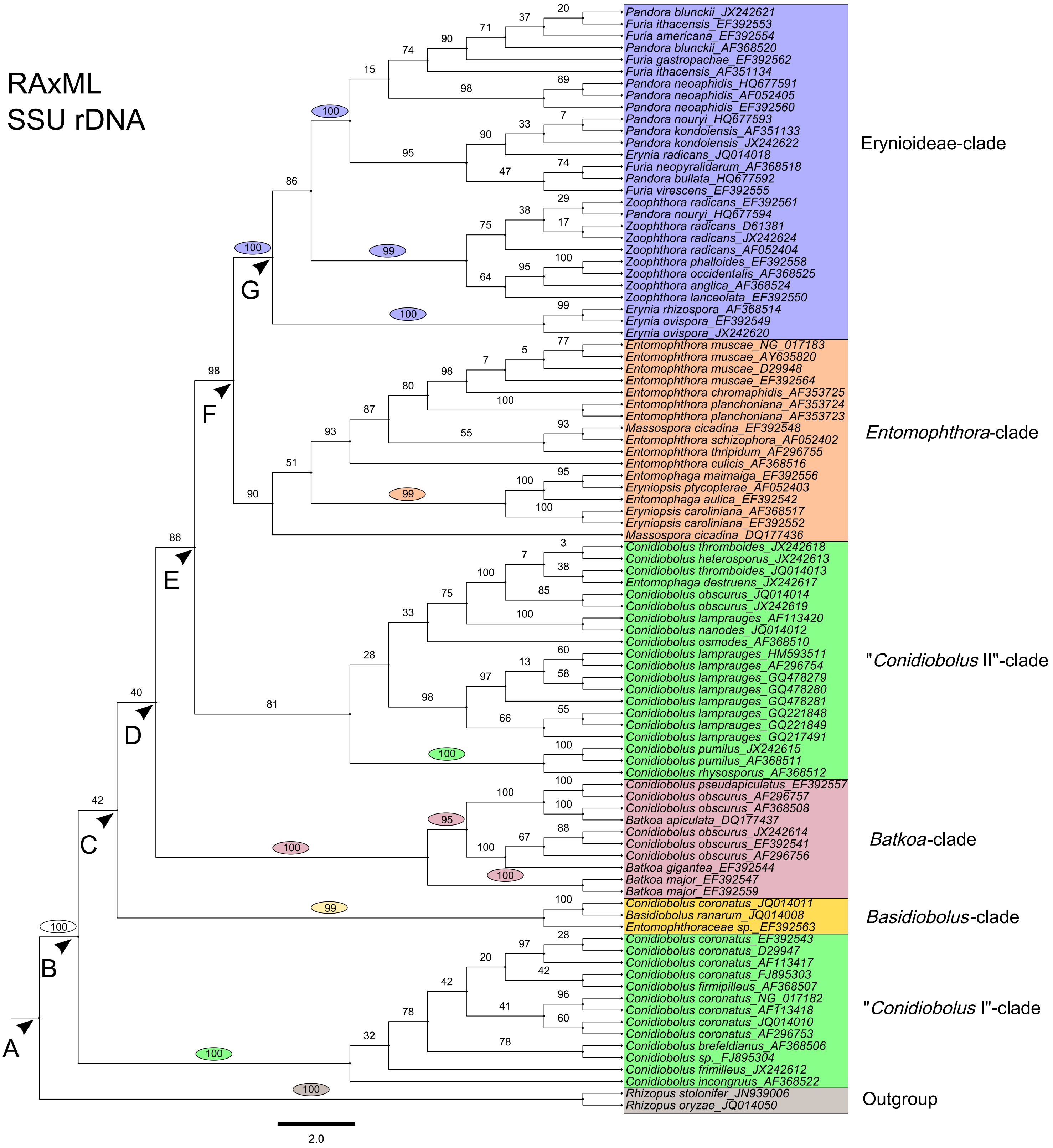

Figure S2: Entomophthoromycotina phylogeny based on a maximum likelihood phylogram reconstruction inferred from large subunit (18S, SSU) ribosomal RNA nucleotide sequences of 91 strains. High, well, and moderate statistical bootstrap support (BS, inferred with RAxML in percentages) is indicated above branches in the color code of the corresponding clade where applicable as in Fig. 3. (A) Split of the outgroup from the Entomophthoromycota lineage, (B) Split of the "*Conidiobolus* I"-clade, (C) Split of the *Basidiobolus*-clade, (D) Split of the *Batkoa*-clade, (E) Split of the "*Conidiobolus* II"-clade, (F) Split of the *Entomophthora*-clade, (G) Split of the Erynioideae-clade. Scale bar indicates substitutions per site.

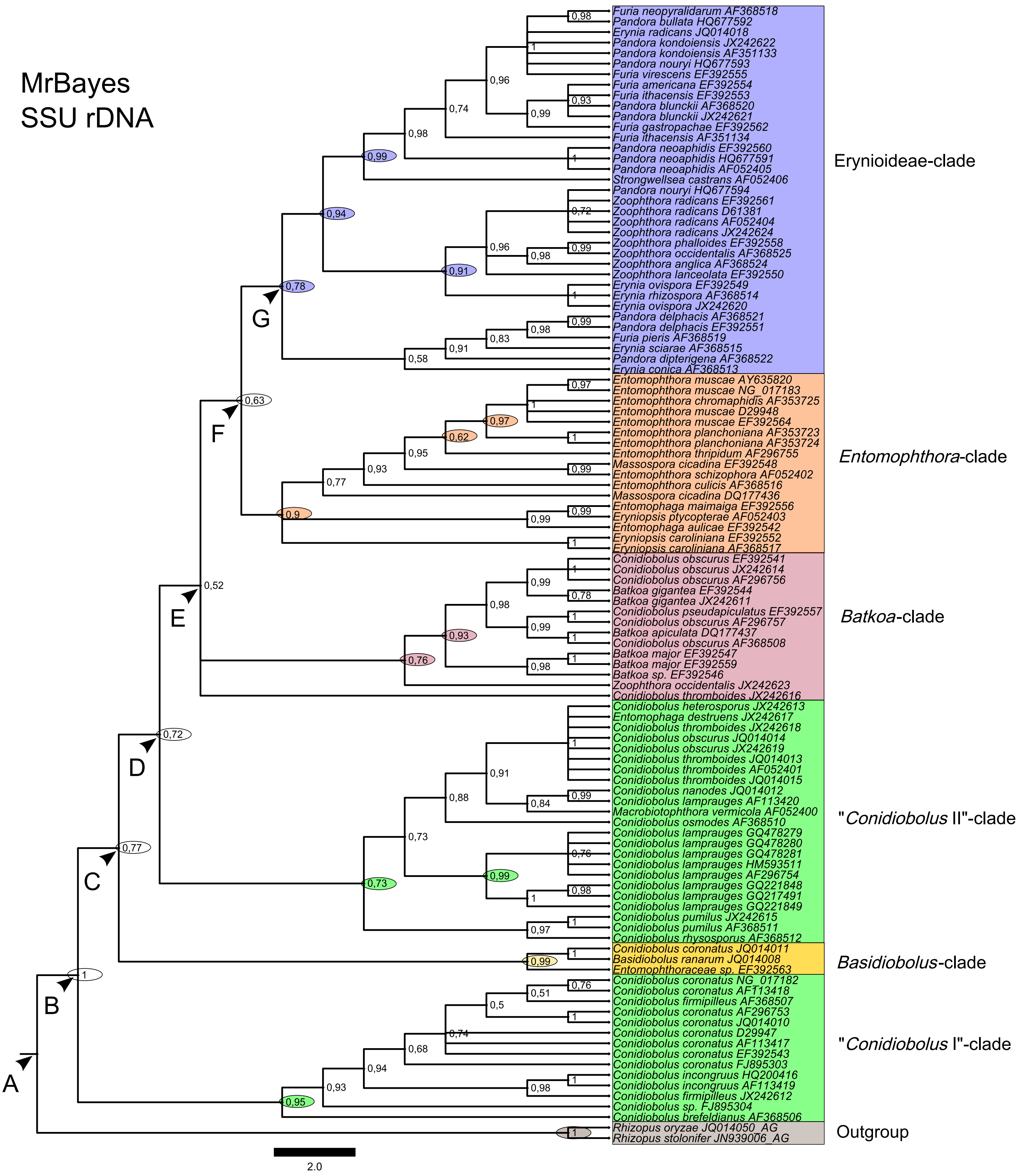

Figure S3: Entomophthoromycotina phylogeny based on a bayesian phylogram reconstruction inferred from large subunit (18S, SSU) ribosomal RNA nucleotide sequences of 91 strains. High, well, and moderate statistical bootstrap support (BS, inferred with MrBayes on a scale from 0 to 1) is indicated above branches in the color code of the corresponding clade where applicable as in Fig. 3. (A) Split of the outgroup from the Entomophthoromycota lineage, (B) Split of the "*Conidiobolus* I"-clade, (C) Split of the *Basidiobolus*-clade, (D) Split of the *Batkoa*-clade, (E) Split of the "*Conidiobolus* II"-clade, (F) Split of the *Entomophthora*-clade, (G) Split of the Erynioideae- and *Entomophthora*-clade. Scale bar indicates substitutions per site.

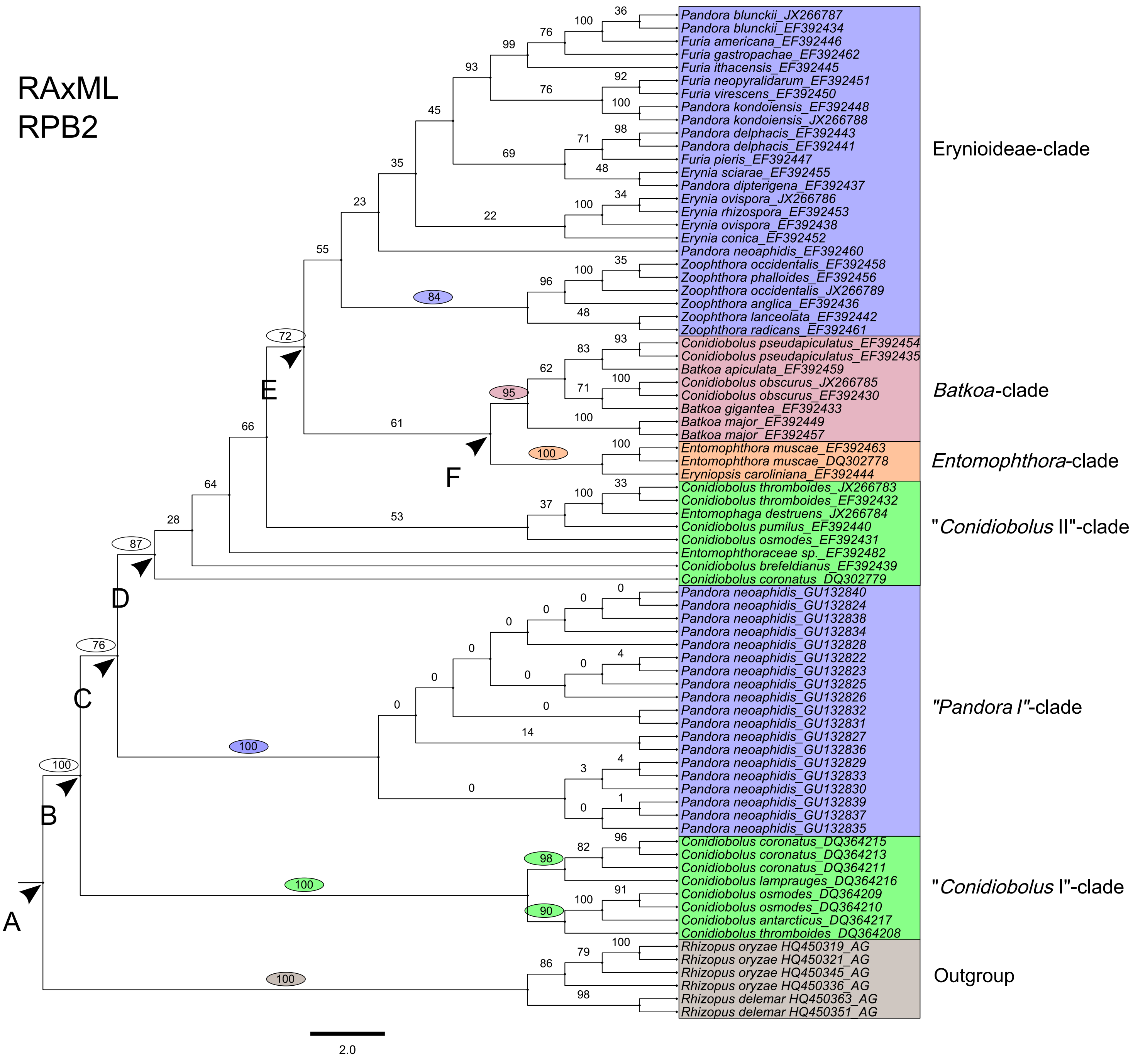

Figure S4: Entomophthoromycotina phylogeny based on a maximum likelihood phylogram reconstruction inferred from DNA-directed RNA polymerase II subunit (RPB2) sequences of 71 strains. High, well, and moderate statistical bootstrap support (BS, inferred with RAxML in percentages) is indicated above branches in the color code of the corresponding clade where applicable as in Fig. 3. (A) Split of the outgroup from the Entomophthoromycota lineage, (B) Split of the "*Conidiobolus* I"-clade, (C) Split of the "*Pandora* I"-clade, (D) Split of the "*Conidiobolus* II"-clade, (E) Split of the *Batkoa-, Entomophthora-* and Erynioideae-clade, (F) Split of the *Entomophthora-* and *Batkoa*-clade. Scale bar indicates substitutions per site.

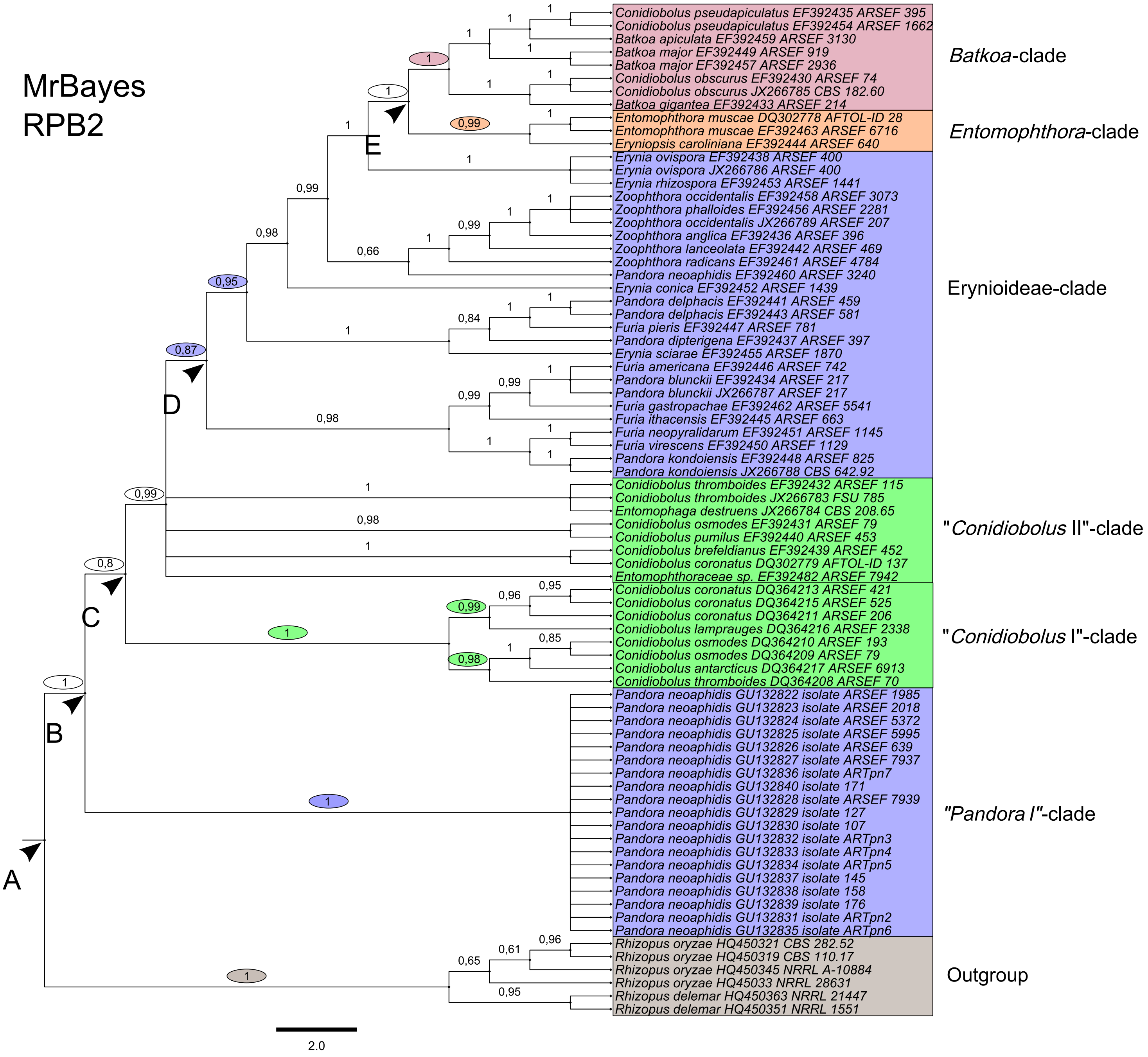

Figure S5: Entomophthoromycotina phylogeny based on a bayesian phylogram reconstruction inferred from DNA-directed RNA polymerase II subunit (RPB2) sequences of 71 strains. High, well, and moderate statistical bootstrap support (BS, inferred with MrBayes on a scale from 0 to 1) is indicated above branches in the color code of the corresponding clade where applicable as in Fig. 3. (A) Split of the outgroup from the Entomophthoromycota lineage, (B) Split of the"*Pandora* I"-clade, (C) Split of the *"Conidiobolus* I"- and "*Conidiobolus* II"-clade, (D) Split of the Erynioideae-clade and *Batkoa-Entomophthora*-clade, (E) Split of the *Entomophthora*- and *Batkoa*-clade. Scale bar indicates substitutions per site.
